# Wearing hats and blending boundaries: harmonising professional identities for clinician simulation educators

**DOI:** 10.1186/s41077-022-00229-w

**Published:** 2022-10-27

**Authors:** William Dace, Eve Purdy, Victoria Brazil

**Affiliations:** 1 Gold Coast University Hospital Emergency Department, Southport, Queensland Australia; 2 Faculty of Health Sciences & Medicine, Bond University, Gold Coast, Queensland Australia

**Keywords:** Clinician simulation educator, Medical education, Professional identity formation, Experiential learning, Phenomenology, Reflection, Sociological fidelity, Psychological safety

## Abstract

Many clinicians working in healthcare simulation struggle with competing dual identities of clinician and educator, whilst those who harmonise these identities are observed to be highly effective teachers and clinicians. Professional identity formation (PIF) theories offer a conceptual framework for considering this dilemma. However, many clinician simulation educators lack practical guidance for translating these theories and are unable to develop or align their dual identities.

An unusual experience involving the first author’s suspension of disbelief as a simulation facilitator sparked a novel reflection on his dual identity as a clinician and as a simulation educator. He re-framed his clinician and simulation ‘hats’ as cooperative and fluid rather than competing and compartmentalised. He recognised that these dual identities could flow between clinical and simulation environments through leaky ‘blended boundaries’ rather than being restricted by environmental demarcations.

This personal story is shared and reflected upon to offer a practical ‘hats and boundaries’ model. Experimenting with the model in both clinical and simulation workplaces presents opportunities for PIF and alignment of dual identities. The model may help other clinician simulation educators navigate the complexities of merging their dual identities.

## Introduction

Clinicians working in medical education can struggle to manage their dual identities of clinician and teacher [1–9], facing individual and social relational [1, 4, 10–16] barriers inhibiting their professional identity formation (PIF) [17]. PIF is the complex dynamic process by which a clinician journeys from ‘who they are’ to ‘who they wish to become’. It has been extensively described in medical students [17–23] but is suggested to be under-theorised in clinician educators [1]. Moreover, few existing theories are adequately aligned with practice to be practically useful [1, 2, 8, 9] for both clinician educators and those with responsibilities for developing these educators. Clinician educators with merged identities are observed to be highly motivated and effective teachers [1, 4], but how they catalyse identity alignment remains incompletely understood and untranslatable for those navigating a messy transition. Without helpful conceptual frameworks or practical guidance, clinician educators risk stumbling through their developmental opportunities and misaligning their identities to clinical or educational contexts.

Many researchers [1, 5–7, 24–29] and organisations [30–36] strategise to encourage clinician educator PIF directly or indirectly, for example by mandating teaching or reflection. But this approach may only be productive for clinician educators operating in amenable environments that address wider social relational PIF barriers [9, 16, 37–43]. Time-pressured clinician educators [9] are cognizant of the importance of teaching and reflection in clinical practice but struggle to mindfully navigate PIF in the face of ever-increasing operational demands [5, 14, 44–48]. These self-fulfilling theory-practice [9] and organisational support gaps [1, 10, 37] suppress the ability and motivation of developing clinician educators to engage in PIF. Change—the process of PIF itself—is often perceived as a threat because of identity ambiguity and misalignment [2, 5, 49]; perceived identity competition and hierarchy are encouraged when clinician and educator identities are compartmentalised in time and space, marginalising the educator identity [2, 4, 5, 7, 11, 14, 50–52].

Popular culture may offer another way of thinking about our competing identities and ways of thinking as clinicians and educators. Based on Edward de Bono’s book *Six Thinking Hats* [53], the idea of ‘putting on a hat’ when switching perspectives has become common parlance. If someone ‘wears many hats’, they have different roles or tasks to perform. De Bono’s work encouraged *teams* to work productively together, through team members assuming different coloured hats. For example, one team member is allocated a white hat and asked to consider ‘facts and information’, while a green hat is charged with thinking of ‘new ideas’, and a red hat to consider ‘feelings and emotions’. De Bono suggested our normal thinking process is a problematic tangle of different types of thinking, but we can improve our decisions by untangling them. This original model has been adapted by educators and coaches for *individuals* to consider different approaches to problems. Given the concept has become well known, it may be accessible for budding clinician educators when considering their dual roles and identities.

Supporting clinician educators to navigate their dual identities is important for them, their learners, and their patients [5]. As the gap between healthcare demand [54, 55] and supply of medical [56, 57] and medical education [4, 58] staff enlarges, we must harness the power of clinician educators. Providing them with simple, practical models of PIF that support their development as both clinicians and educators may be one step in that process. In this article, we present a new ‘hats and boundaries’ model of PIF development for clinician simulation educators.

### What this article adds

- Reframes simulation and clinician identities as cooperative (rather than competitive) and fluid (rather than compartmentalised), modelled by the clinician and simulation hats flowing through ‘blended boundaries’ into the simulation and clinical environments, respectively.
- Through this model, reframes the challenge of clinician simulation educator PIF as a continuous, bidirectionally leaky process, presenting an opportunity for developing and aligning dual identities rather than a threat.
- Provides practical examples of opportunities the model offers for clinician simulation educators and learners.

## Developing the ‘hats and boundaries’ model through reflection

This model was developed from structured reflections of the first author’s (WD) experience as a simulation facilitator and clinician educator over a 3-month period. Our approach draws on the phenomenological traditions—the study of a phenomenon to understand an experience from those who have lived it [59]. While usually performed to ‘others’, there is increasing application to use structured reflection to study the researcher’s own experiences [60]. WD is an emergency department senior house officer and simulation educator at Bond University. During a 3-month period between February and April 2022, he wrote a series of reflections related to PIF (over 20,000 words) and engaged in ongoing reflective verbal and written communications with VB and EP. This series of reflections and communications served as the basis for the development of a new model for PIF for clinician educators.

The PIF model derived is directly drawn from the experience of the first author, and so *script in italics* is subsequently written from a first-person singular perspective. Regular text in the rest of the article is written from our collective perspective. WD is a resident with an evolving interest in education, early in his PIF as a clinician and educator. EP is an early career emergency physician, educator, and researcher who is further along than WD in navigating PIF but still early in this journey. VB is an emergency physician and experienced educator who has supervised many professionals as they develop dual identities. As such, our team is well positioned to reflect on the process and potential utility of the model we present.

### The Bond Simulated ED Exercise as a catalyst for model creation

The Bond University medical programme includes a large-scale simulation-based education (SBE) exercise as part of the preparation for final-year students for internship [61]. The faculty comprises a team of real emergency department (ED) doctors and nurse facilitators, adopting their normal clinical roles. Students take on their future roles as ED interns and work a continuous 2-h shift in a simulated ED. The ED has a capacity for 9 simulated patients, with the arrival of new patients after every discharge. Significant efforts are made to re-create the busy environment of the ED, with realistic tasks and competing priorities for simulation participants and facilitators. Below, WD reflects on his dual identities as an educator and clinician at one Bond simulated emergency department exercise (BSEDE):

Cluttered with the distractions found in real clinical work, the Bond simulated emergency department exercise (BSEDE) environment and tasks felt less scripted and sterile. The BSEDE subscribed to synchrony of functional task alignment, physical resemblance [62], and sociological fidelity [63] with the real workplace role, environment, and interactions. Participating facilitated my true suspension of disbelief for the first time as a simulation educator. I entered a liminal space with ‘blended boundaries’ between how I thought, felt, and acted - my professional identity [17] - as a clinician and as a simulation educator. This new, ambiguous state of being felt unusual; I previously considered these dual identities as compartmentalised, with their separation reinforced by the different environments, operational demands, interactions, and time pressures associated with each role. Reflecting after the BSEDE, I realised this feeling of my identities blending stemmed from my usual educator focus shifting away from “teaching” and towards caring for the simulated patients.

I had recently read Purdy’s concept of ‘leakiness’ to model flow of psychological safety between the simulation and clinical environments [64]. Whilst reflecting, I considered whether dual clinician and simulation educator identities could flow similarly between these environments. I seemed to experience this leakiness during the BSEDE when thinking and behaving as if engaged in real clinical work, despite maintaining awareness of my educational role and physical presence in the simulation environment. I started to think about clinician and simulation educator ‘hats’, inspired by de Bono’s Thinking Hats [53], a conceptual thinking framework to which I was introduced as a schoolchild.

### Wearing hats and blending boundaries

Drawing on de Bono’s positive framing of different ways of thinking, the hats and boundaries model can reframe dual identities as cooperative and fluid rather than competing and compartmentalised in the clinical and simulation environments in which they typically respectively predominate.

**Fig. 1:**
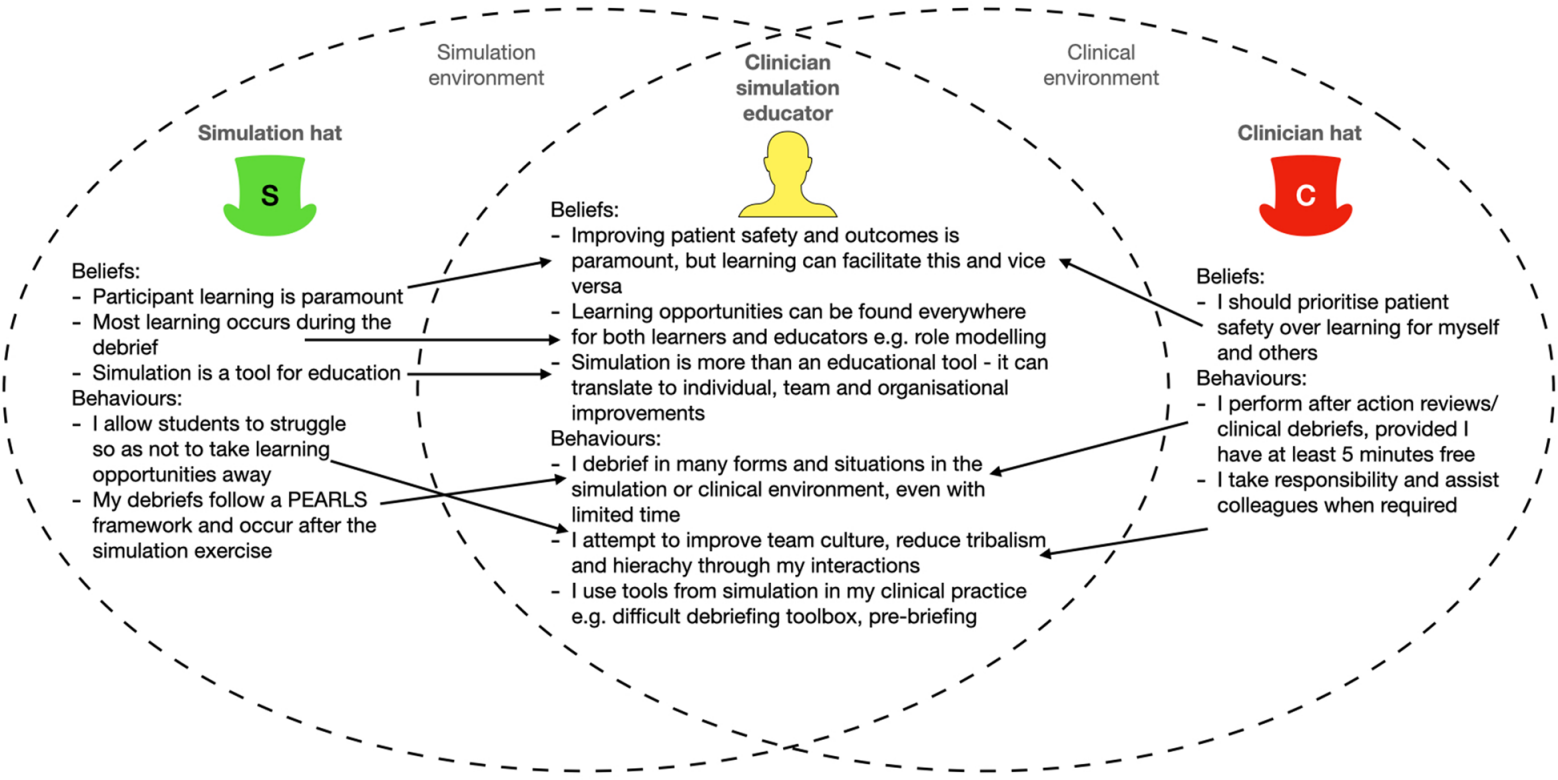
Adopting the clinician hat in the simulation environment (and vice versa), permitted through leaky blended boundaries, can reshape existing beliefs and behaviours associated with clinician and simulation identities. Arrows represent the flow of WD’s hats and associated pre-existing beliefs and behaviours into the other environment. The Venn diagram intersection represents WD’s new harmonised professional identity and associated new beliefs and behaviours

The clinician and simulation educator hats represent the two different clinician and simulation identities usually adopted by clinician simulation educators in the clinical and simulation environments, respectively. For example, one can don the clinician hat by asking oneself: ‘how would I respond to this situation in the clinical environment?’ In the model, unrestricted by environmental demarcations, the hats flow through leaky ‘blended boundaries’ between clinical and simulation environments to form a harmonised professional identity (Fig. 1). This model could reshape and align dual identities and highlight PIF opportunities for clinician simulation educators and learners.

Below WD provides a more in-depth example of these blended boundaries and harmonised identity in practice:

Reflecting on my BSEDE experience clarified that this feeling of ‘blended boundaries’ stemmed from natural switching to my clinician identity during certain scenarios. I questioned whether I was wearing my ‘clinician hat’ (‘what would I say or do in clinical work?’) or ‘simulation hat’ (‘what would I say or do in simulation?’) in each situation.

For example, during the BSEDE when a student asked for help interpreting an electrocardiogram (ECG) clearly showing an ST-elevation myocardial infarction (STEMI), I unconsciously donned my clinician hat by declaring this was a STEMI and instructing her to call the interventional cardiologist - rather than helping her interpret the ECG first; a simulation educator behaviour I have applied in previous emergency simulations motivated by my simulation belief: ‘simulation is a tool for education’. On reflection I realised this authentic clinical behaviour, driven by my clinician belief that ‘patient safety takes priority over learning’, provided learning through role modelling of crisis resource management skills [63]. After the simulated patient left ED, we discussed the ECG during a two-minute debrief whilst the exercise continued around us, driven by my simulation educator belief that ‘most learning occurs in the debrief’. My dual identities cooperated rather than quarrelled. Through reflection I formed the new beliefs that learning opportunities are everywhere, and even short mid-exercise debriefs can reinforce them (Fig. 1). I now make conscious effort to don my clinician hat in simulation scenarios and debriefs as part of my simulation identity.

This ‘leak’ of the clinician identity into the simulation space is not a new concept. Our research [64] team has previously demonstrated that participants’ clinical roles and prior team interactions ‘leak’ into the simulation space, impacting the way that they engage with simulation. WD’s reflection shows that this is also true for facilitators. Awareness of how clinician identity impacts the role as a simulation facilitator—how that hat ‘leaks’ into simulation for better or worse—is a powerful and important step in PIF. Below WD further explains the power of this clinician hat leaking into simulation:

In another example from the BSEDE, during an end-of-life discussion with a dying simulated patient’s family, the student leading the discussion was unable to answer a question and looked at me as if to say ‘help’. I stepped in to answer, driven by my clinician identity attitude to take responsibility and assist colleagues, before handing the discussion back over to the student. On reflection I realised the high fidelity of task and sociologic context had shifted my internal identity from ‘teacher’ (instructing learners - simulation identity) to ‘more experienced doctor’ (on the same team as new doctors - clinician identity). Without my immersion in the simulated family room equipped with a coffee table, potted plants, and distressed daughter, I may have allowed this student to struggle. My simulation educator belief that ‘learning is paramount’ (combined with previous unawareness of psychological safety’s importance in learning) has previously motivated this behaviour in similar scenarios. I experienced the ‘let them learn’ attitude from simulation educators as a struggling medical student, leading to embarrassment and the deleterious consequences of reduced psychological safety [64, 65].

Wearing a clinician hat may also have altered learner perceptions of faculty identity and may have fostered stronger team orientation than often experienced in educational settings.

Students seemed more comfortable asking for help compared to my previous facilitating experiences. I attributed this to the simulation design and my clinician hat use increasing the sociological fidelity [63] of our interprofessional interactions; students experienced me proactively offering help and my language tended towards collective nouns (‘we’, ‘our’), away from my educator language (e.g. ‘you guys’) that can reinforce hierarchical boundaries. The resultant rapid cultivation of supportive team culture and psychological safety I perceived was striking as ‘the team' had not previously met and multiple scenarios were designed to be high-stress for students.

Learner ‘suspension of disbelief’ in SBE is encouraged by facilitators when trying to maximise engagement within the simulated environment. This is often described as a ‘fiction contract’ between simulation faculty and learners [66]. However, suspension of disbelief as a simulation educator may be part of the process of harmonising dual identities.

The BSEDE’s close physical resemblance and functional task alignment [62] with my real clinical workplace and role facilitated my initial suspension of disbelief and natural switching to my clinician hat during the exercise. Deliberately maintaining my clinician identity during mid-BSEDE debriefs focused my attitude and language on how our team could improve patient care. I realised this helped naturally generate advocacy statements [67] and ‘safe not soft’ debriefing [68]. Because this patient-centric debriefing reduced my fear of upsetting learners with feedback - a recognised tension between dual identities [5] - the clinician hat improved my confidence delivering psychologically safe mid-scenario debriefs. Post-BSEDE reflection prompted comparison of existing beliefs and behaviours of my dual identities, leading to the formation of a new professional identity (Fig. 1). Cooperation (rather than competition) between fluid (rather than compartmentalised) dual identities facilitated my PIF.

## Applying the model

### The habit of switching hats

These reflections suggest there are benefits in intentionally adopting a clinician hat as part of simulation identity during other SBE scenarios and debriefs.

**Fig. 2:**
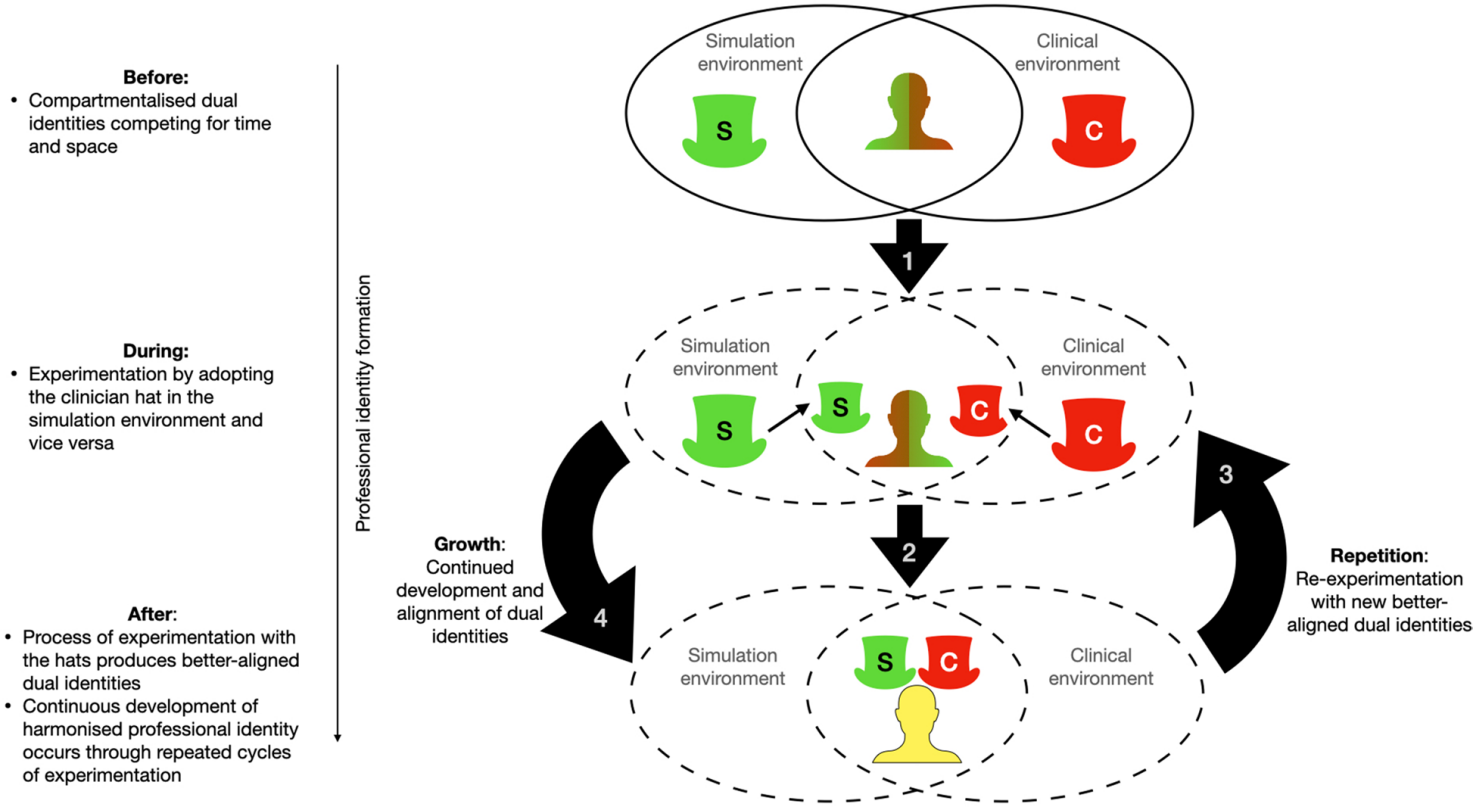
The continuous process of professional identity formation by experimentation with the clinician and simulation hats through blended boundaries

Repeated use of my clinician hat in subsequent simulation exercises has catalysed new iterations of my simulation educator identity influenced by useful beliefs, values, attitudes and behaviours from my clinician identity (Fig. 2). For example, deliberately switching hats now provides clarity for my decision-making during simulation interactions. With awareness of the potential profound ripple effects from seemingly small interactions [64], this habit plays an important role in increasing the authenticity of my communication in simulation and potentially bridges the sociological fidelity gap for learners in SBE [63] as described in the BSEDE examples. This habit has also reduced the influence of physical and functional fidelity of simulation design [62] on my ability to suspend disbelief; actively choosing to adopt my clinician identity (and the resultant realistic ‘feel’ of interactions) has become sufficient for my immersion in simulation. Assisting simulation learners whilst wearing my clinician hat also serves as easily translatable ‘practice’ for when I supervise other clinicians in the clinical environment.

Through the hats model of PIF, my clinician identity flows through blended boundaries into the simulation environment to support development of my simulation identity, bringing my dual identities increasingly into alignment. PIF provides an opportunity for growth rather than threat to my identity.

And perhaps, could this intentional ‘hat switch’ be applied in the other direction?

I wondered if my simulation identity could similarly influence how I thought, felt, and acted in my real clinical work. Using the model to guide reflection, through experiential learning I found that wearing my simulation hat in the clinical environment could reshape my clinician identity through the same blended boundaries.

For example, an attempt to calm down an angry intoxicated patient on a (real) ED nightshift prompted me to don my simulation hat and approach the situation like a difficult debrief, taking inspiration from Grant's toolbox to elicit the reasons for his anger, empathise and respond appropriately (Table 1). I now consciously don my simulation hat to use the adapted version of this toolbox as a clinician when breaking bad news, dealing with conflict and incivility [69, 70], and during other difficult conversations.

**Table 1:** Adapting the difficult debriefing toolbox for clinical use (adapted from [71])

Strategy	Definition	Objective	Phrase I used
*Naming the dynamic*	Drawing out a ‘hot topic’ by explicitly naming it	Focus discussion on ‘hot topic’	‘I sense some anger, is there anything contributing to this that I can help with?’
*Validation*	Acknowledging that patients’ feelings, behaviours or thoughts are acceptable	Reaffirming patients’ perspective	‘You’re right, waiting is frustrating, especially when you’re in pain.’
*Paraphrasing*	Restating something in your own words	Clarify understanding	‘If I’ve understood correctly, you’re upset because being here in hospital has reminded you of anxiety-provoking experiences from the past?’
*Normalisation*	Relating behaviours, feelings or attitudes to a societal norm	Build trust, calm fear, defuse emotions	‘It’s understandable for you to feel anxious in the emergency department. I’m sure other patients share these feelings tonight; it is busy and noisy.’
*Previewing*	Introducing a new topic to discuss	Focus patients on a topic of conversation	‘Why don’t we switch gears and discuss how best to treat your injuries so we can get you home sooner.’

### Opportunities for clinician simulation educators

#### The hats as a reflection tool

Organisational strategies to encourage clinician educator reflection, such as faculty development programmes [5], workshops [25] and video-reflexive ethnography [24] are important but risk providing diminishing returns for clinician educators already under pressure [4, 10, 14, 26] to ‘prioritise clinical responsibilities’ [5]. Clinician educators must tailor their ‘own programme on professionalism’ to plug the theory-practice gap [9], but lack simple reflective tools to help with that process. Frictionless tools for self-sufficient reflection are essential considering the lack of defined education, roles, or training pathways [4, 5, 9, 58, 72] for clinician educators.

I remember struggling to generate reflections as a foundation doctor, cramming this mandatory task [31] in at the end of clinical rotations. Using the model, my personal process of PIF involves simple comparison of my dual identities to generate novel reflections; when faced with a problem in my clinical workplace, I can ask myself ‘how would I approach this in the simulation environment?’ (or vice versa) either in real-time or after the event.

#### Translating simulation tools into clinical tools

Clinicians could translate simulation tools into the clinical environment by experimenting with their simulation hats. For example, the difficult debriefing toolbox discussed above [71] was designed to empower simulation educators to recognise different phenotypes of difficult debriefing situations and provides strategies to address them. These same tools can often be useful in clinical practice. Other tools and concepts commonly employed in simulation such as pre-briefing, debriefing and psychological safety can be adapted to help teams in real clinical practice as WD shares below:

My simulation hat has also helped translate my knowledge and experience of simulation pre-briefing and debriefing into the clinical environment through conduction of shift huddles and after-action reviews - the respective clinical equivalents of these simulation educator skills. These new habits have improved my ability in both environments to flexibly seize opportunities to pre-brief and debrief regardless of the situation.

### Opportunities for simulation learners—clarity and growth

Simulation educators can mistakenly assume effective information transfer to learners [66]. Discussing the hats can provide clarity for learners, supporting engagement and psychological safety. Note the simulation hat takes on a different meaning of ‘simulation learner hat’ here, rather than ‘simulation educator hat’.

#### Establishing clarity for simulation novice learners

Even with sufficient clinical knowledge, the liminal space of simulation can generate confusion. An explicit reference to ‘changing hats’ could ease the awkwardness of a perceived shift in role or context. WD shares how he used this concept to help simulation learners below:

During a SBE debrief [73], a final-year student told me: “I wasn’t sure how long to wait before putting out a medical emergency team (MET) call because it's simulation, then I got stressed trying to make that decision.” I asked her to remove her simulation hat and next time don her clinician hat - to think and behave like she would as a doctor in the clinical environment. In the next scenario iteration, she activated the MET call appropriately.

**Fig. 3:**
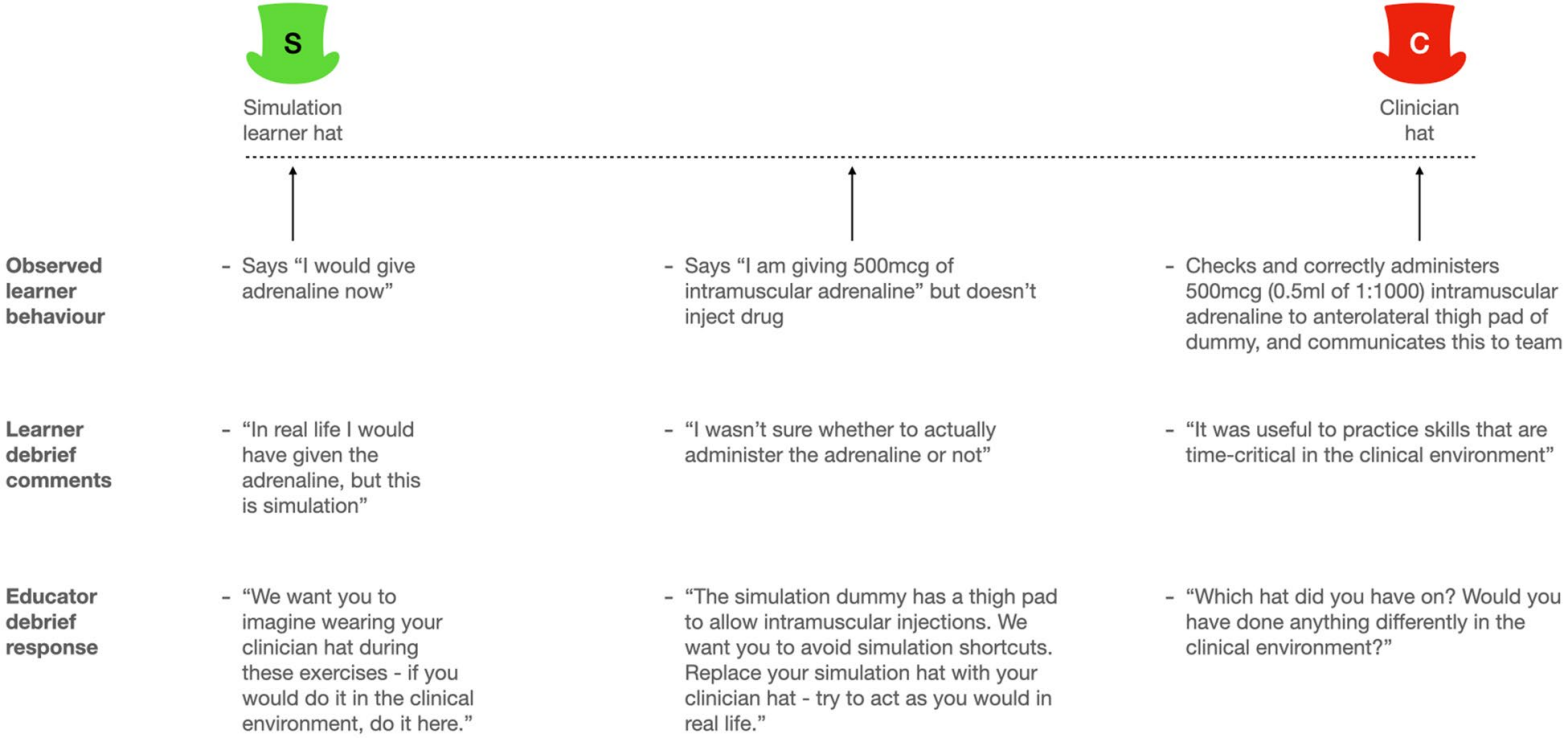
Utilising the hats model to reduce confusion and simulation shortcuts during adrenaline administration in an anaphylaxis medical emergency simulation exercise [74]

Here, the hats model provided a mid-SBE debriefing intervention to clarify educator expectations through the lens of identity. The example in Fig. 3 was generated by revisiting, through the lens of the hats model, WD’s reflections on a previous teaching experience involving learner confusion [74]. It illustrates that by narrowing the focus of the hats model to the performance of time-critical skills, such as adrenaline administration, the model may help reduce confusion and simulation shortcuts [75].

#### Encouraging a growth mindset through the notion of ‘future clinician hats’

The use of the hats might facilitate growth by explicitly acknowledging the personal risk participants take when “stepping up” to attempt a new skill in the simulation environment.

As a learner in a recent paediatric emergency simulation, I intubated a hypoxic 8 month-old baby. It would be neither safe nor expected at my grade to do this in the clinical environment, but the simulation educators gave me permission to attempt this before the task began. To help bridge this performance gap between my current clinician identity and imagined future clinician identity, they could have asked me to test a future aspirational iteration of my clinician hat - an often unspoken expectation we have of medical students in simulation.

Educators can encourage learners to try, and provide permission to fail, by using the hats to acknowledge when we ask learners to adopt their future clinician hat (as in the aforementioned MET call scenario example) [65]. Providing the psychological safety for learners to stress-test their ‘possible selves’ [76] can be linked to a growth mindset [77]—the belief that human intelligence and ability are malleable through purposeful effort—which holds benefits for medical learners and educators [78, 79], including in simulation [80].

## Future directions

PIF is influenced by the relationship between an individual’s personal identity and professional identity [38, 81, 82]. The first author’s *personal* identity was a strong influence on his *professional* identity formation and development of the hats model. This article’s deliberately individualist focus, required to describe the model’s inception clearly, is a simplification because workplace social and contextual factors influence the identities of clinician educators [1]. For example, high clinical workloads undermine educator identity [4, 5]. In the model, the hats are variables representing the clinician educator identities, whereas blended boundaries demarcate the leaky environmental domains that individuals occupy within the cultural milieu of the team and organisational structures. For a truly translational model, future iterations must reflect how these wide-ranging contextual variables influence the leakiness of blended boundaries, with work directed at how this influences the flow of the hats and PIF for embedded individuals.

Examining the model in other clinician educators will be important—e.g. for nurses working as embedded simulation participants in medical student SBE scenarios. This faculty group must rapidly switch roles between participating clinicians and simulation educators during scenarios and likely also experience bidirectional impacts from and back to their clinical roles. Modifications may also be necessary to ensure the model is both inclusive and adaptable—especially for clinicians and students underrepresented in medicine, including clinicians with minoritised ethnic backgrounds who must negotiate challenges to PIF differing in scale and nature from those faced by non-minoritised groups [19, 38, 81, 83–86].

## Conclusion

The Latin origin of ‘doctor’ is *docēre*, meaning ‘to teach’. Prompted by the experience and reflections of the first author as a simulation facilitator, we suggest reconceptualising the roles of clinician and educator through blended, leaky boundaries. Habits of reflection and intentionally ‘switching hats’ can harmonise dual identities for clinician educators and provide conceptual guidance for future practice. There are opportunities for faculty development in medical education to redirect focus, moving away from PIF professionalisation toward addressing wider social relational barriers and promoting clinician educator PIF through our practical model.

## Data Availability

Not applicable.

## Abbreviations

PIF: Professional identity formation
SBE: Simulation-based education
ED: Emergency department
BSEDE: Bond-simulated emergency department exercise
ECG: Electrocardiogram
STEMI: ST-elevation myocardial infarction
MET: Medical emergency team
